# China's goal of achieving carbon neutrality before 2060: experts explain how

**DOI:** 10.1093/nsr/nwac115

**Published:** 2022-06-16

**Authors:** Weijie Zhao

**Affiliations:** NSR news editor based, Beijing, China

## Abstract

‘We aim to have CO_2_ emissions peak before 2030 and achieve carbon neutrality before 2060,’ President Xi Jinping so declared at the General Debate of the 75th United Nations General Assembly on 22 September 2020. More than 130 countries globally have proposed their own carbon neutrality goals by 2050 or 2060. Thus, carbon neutrality is a collective effort of human societies to cope with the climate crisis. If all countries could follow their own plans and reach carbon neutrality in a few decades, we may have a chance to control global warming within 1.5 or 2^o^C, confining climate change to a relatively safe zone. As a developing country with a large population, high coal consumption and large manufacturing industries, can China achieve the huge task of societal transformation that will enable carbon peaking and carbon neutrality within the next four decades? How will China transform traditional power generation and manufacturing industries, as well as create new technologies for carbon capture and storage? In this panel discussion chaired by Prof. Xinhe Bao, a scientist of energy and chemistry, top experts gathered to discuss the challenges and potential solutions, outlining the coming ‘green industrial revolution’.

Huiming Cheng

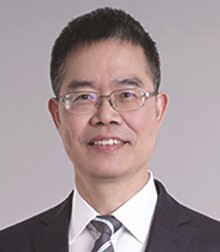

Professor, Institute of Metal Research, Chinese Academy of Sciences; Shenzhen Institute of Advanced Technology, Chinese Academy of Sciences

Zhengtang Guo

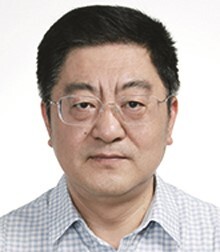

Professor, Institute of Geology and Geophysics, Chinese Academy of Sciences

Yaling He

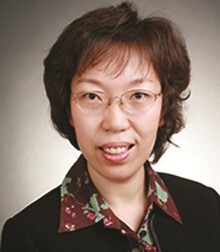

Professor, School of Energy and Power Engineering, Xi’an Jiaotong University

Zheng Li

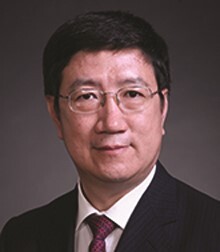

Professor, Institute of Climate Change and Sustainable Development, Tsinghua University

Minggao Ouyang

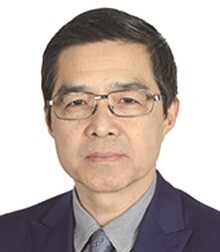

Professor, School of Vehicle and Mobility, Tsinghua University

Zhengrong Shi

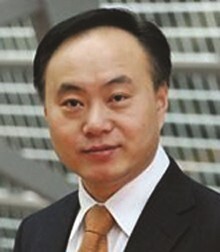

Professor, College of Energy and Mechanical Engineering, Shanghai University of Electric Power

Zaiku Xie

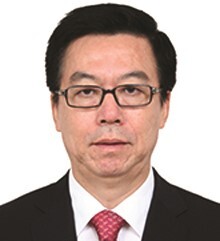

Professor, China Petroleum and Chemical Corporation (SINOPEC)

Xinhe Bao (Chair)

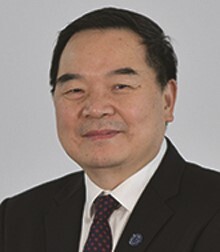

Professor, University of Science and Technology of China, and Dalian Institute of Chemical Physics, Chinese Academy of Sciences

**Bao:** Today we have experts from different disciplines related to energy to talk about the issues of carbon peaking and carbon neutrality in China. Since September 2020, China has initiated many plans and strategies for achieving these goals. I would like to invite Prof. Li to introduce the background and significance of achieving carbon neutrality in China.

**Li** **:** There is now a global consensus on the crisis of climate change. It is a challenge for all human societies. Carbon peaking and carbon neutrality are the solutions targeting the source of climate change—the emission of greenhouse gases. Most countries have promised to achieve carbon neutrality, and from the European Green Deal to China's initiatives, the goal is not just

From the European Green Deal to China's initiatives, the goal is not just to reduce carbon emissions, but also to initiate a ‘green industrial revolution’.

—Zheng Li

to reduce carbon emissions, but also to initiate a ‘green industrial revolution’ and lead human society to a new path that does not depend upon fossil energies. Going green has become a global trend. If China wishes to achieve sustainable development in the future, the only wise choice is to follow, or even lead this trend. So, we can see that setting the carbon neutrality goal is a well-thought-out strategic decision of the Chinese government.

Almost all countries aim to achieve carbon neutrality, but each country has its own energy structure and development stage, so the degree of difficulty in achieving these goals is different. In fact, western developed countries have already reached peak carbon emissions. Europe and the US reached their peak in the 1980s and 2005, thus have ∼70 and 45 years to achieve neutrality by 2050, respectively. But China is still developing and has not reached its peak. Moreover, our industrial structure is biased towards heavy industry and our energy structure is biased towards coal use, so it is a great challenge for China to reach its peak by 2030 and achieve neutrality by 2060.

Furthermore, not all countries are equally responsible for the current climate crisis. Developing countries have not emitted as much carbon, historically, as developed countries, and they also have the right to develop. At the Glasgow Climate Change Conference in 2021, developed countries proposed setting 1.5°C, instead of 2°C, as the only limit for temperature rise, and proposed that all nations should ‘phase out’ the usage of coal. After negotiations and compromises, the final document kept the 2°C limit set by the 2015 Paris Agreement, and pointed out that we should keep 1.5°C within reach. It also replaced the term ‘phase out’ with ‘phase down’ regarding coal use.

To summarize, to achieve carbon neutrality and to create a green society is a significant task for the entire world, benefiting all humanity.

**Bao:** To achieve carbon neutrality, what can we learn from other countries?

**Li** **:** There was a study of Net-Zero America in the US, which presented six different future scenarios under different energy structures and electrification levels. The study proposed several major strategies that we can learn from. First, the electrification of energy consumption end users is the most important approach for decreasing energy consumption and carbon emissions. Second, to achieve carbon neutrality, non-fossil energy will definitely need to become the primary energy source, but some fossil energy has to be maintained for the stability of the power grid. Thus, keeping a proper ratio of fossil and non-fossil sources is important. Third, carbon capture and storage (CCS) technologies must be simultaneously developed to neutralize the carbon emitted from fossil fuel usage. Actually, the CCS capacity is the decisive factor in determining the amount of fossil fuels we can use.

**Guo**: Because of different social conditions, the challenges facing each country are also different. Many strategies cannot be simply transferred from other countries to China. I think there are three things we can learn from advanced countries. First, we can adopt advanced green technologies already implemented abroad. Second, we can learn how to build a low-carbon culture from European countries. In many European countries, almost everybody accepts the low-carbon concept, preferring to buy small-engined cars and saving energy and resources in their daily lives. That is what we can strive for. Third, in addition to the comprehensive national policies we have initiated, we should make customized plans of carbon neutrality for each province, each city and each town, in accordance with the unique regional conditions of natural resources, development level and energy structure.

**Cheng**: When we talk about carbon neutrality, we mostly focus on energy and resource, but in Europe, people also consider industry, architecture, transportation, agriculture, ecology and many other sectors of society. I think this systematic approach is what we can learn from.

**Bao**: China is also considering these various approaches. But as energy-related researchers, we may hear more about topics on energy. But you are right that carbon neutrality is a systems problem. Maybe we should first sort out the major topics we could talk about.

**He**: I think there are three major aspects: first, emissions reduction in energy generation, namely, gradually replacing fossil fuels with natural gas and renewable energies; second, green transformation of energy consumption, such as the transformation of industries and transportation; third, development of CCS technologies. Moreover, both energy generation and energy consumption sectors are calling for breakthroughs in energy storage technologies.

## POWER GENERATION: GOING GREEN

**Bao**: Coal has been the primary energy source in China. Can we further improve the efficiency of coal-fired power plants, to generate more electricity with less coal?

**He** **:** Yes. Currently, about half of China's coal-fired-power-generating units are advanced supercritical or ultra-supercritical units, with a main steam temperature of 570–612°C, energy efficiency of 40%–45% and carbon emissions per kilowatt-hour of 700–800 grams. If we can further improve the ultra-supercritical units, the steam temperature is likely to reach 700°C, with the efficiency increasing to 50% and carbon emissions decreasing to ∼640 grams per kilowatt-hour.

Electric power sources will definitely transfer from fossil fuels to renewable energies. The role of coal will change from a primary source to a supportive one. On the one hand, solar

The role of coal will change from a primary source to a supportive one.

—Yaling He

energy and wind energy are not stable, so we need coal plants to ensure the stability of power supply. On the other hand, technologies combining fossil and non-fossil sources are fast developing. As examples, photothermal power generation technologies and molten salt heat storage technologies can be integrated into the current coal-fired units to reduce the consumption of coal; also, energy storage systems and heat pump units can be coupled with renewable power plants to reduce solar and wind power curtailment. In short, multiple reforms are happening in coal-related industries to transform the role of coal in the energy system.

Importantly, energy storage plays a key role in many fields, including new energy, multi energy coupling and power-consumption devices. The realization of low-cost high-safety large-scale energy storage technology will make critical contributions to the ongoing energy revolution.

**Bao:** Thanks. Prof. Shi is one of the pioneers of China's solar photovoltaic industry. Would you please summarize the recent development of this field?

**Shi:** The photovoltaic industry has been developing fast in the past two decades. Here in China, we have advanced technologies, strong teams and the whole supply chain. Currently, >90% of the parts in the supply chain are regularly made in China. China's photovoltaic power output has ranked first in the world for 15 consecutive years. This fast development is a result of strong support, both from the government and from private investments.

The boom of the photovoltaic industry laid a solid foundation for China to achieve carbon neutrality by 2060. Globally, with policy support, the photovoltaic electricity price has dropped to a level competitive with fossil-fuel electricity. By the end of 2021, photovoltaic capacity had reached 1 terawatt (TW) globally and 0.3 TW in China. To achieve the carbon reduction goals set by each country, the capacities need to increase by 2 TW globally and 0.8 TW in China by 2030—in less than 10 years’ time. So the question is: can the photovoltaic industry expand at such a rapid pace? Technically, the major element of the photovoltaic industry is silicon, which is the second richest element on Earth, so there will not be a scarcity of raw materials. But with the new round of large-scale investments and development, the supply chain grew out of balance; the production speed of the upstream polysilicon is much slower than that of the downstream solar cell modules, so the price of photovoltaic modules has risen by ∼20% since 2021. We have to cope with this issue for further expansion of this industry.

Regarding future directions, I think distributed generation has great potential. China is preparing to build large centralized photovoltaic farms in western China to provide

0.15–0.2 GW capacity. At the same time, distributed photovoltaic generation is also developing fast, especially in provinces that have good sunshine conditions, such as Shandong, Anhui, Henan and Hebei, where the distributed capacity reached 40% of total photovoltaic capacity in 2021. Many residents are happy to rent their roofs for distributed generation and earn some money from that. Another example is that some internet companies are planning to build large data centers in eastern China. In order to meet the government's requirement of carbon reduction, these companies intend to construct photovoltaic plants in the west, but the problem is how to transfer the green electricity generated in the west to the east? It cannot be solved by the companies. So my suggestion is that they could construct distributed photovoltaic plants locally in the east, which would be enough to fulfill their needs.

Another trend is to combine photovoltaic technology with many other industries such as building materials, vehicles, agriculture and urban lighting. Particularly, photovoltaic hydrogen production has become a hot field that is attracting a lot of investment. The technology is improving and the price is dropping, proving its potential.

The last thing I want to say is that, basic research in this area is important but the progress is relatively slow. So from the industrial point of view, we should make full use of the existing technologies as soon as possible to achieve carbon neutrality in time. For example, large-scale energy storage is a hard nut to crack for centralized photovoltaic generation, but distributed energy storage is relatively easy, so distributed photovoltaic generation is an appropriate direction for now.

The boom of the photovoltaic industry laid a solid foundation for China to achieve carbon neutrality by 2060.

—Zhengrong Shi

**He** **:** Besides photovoltaic and wind power, which have developed into relatively mature industries, photothermal power is another important kind of renewable energy. China started late in this field and the photothermal industry has not taken off, but the technology has been well developed and we are able to construct the whole system in China. Moreover, photothermal plants can provide not only electricity, but also a long-cycle low-cost large-scale heat storage pathway, and its long industrial chain will also promote related industries.

**Cheng**: I heard a lcture about ‘photovoltaic sheep’ and ‘wind power cattle’, which means that on the land under photovoltaic and wind farms, grass is growing to herd sheep and cattle. Therefore, photovoltaic and wind farms may not damage local ecology, but on the contrary, improve it.

**Bao**: I have actually seen this in western China. The plants use water for equipment cleaning and that water naturally moistens grass. However, sheep and cattle cannot be herded under photothermal plants, because the sheep would gore and tilt the photothermal mirrors, meaning they would be unable to focus sunshine.

**Shi**: Herding sheep and cattle under the power plants has another benefit: it clears off the grass to prevent fire. It actually lowers the maintenance cost of the power plants.

## ENERGY CONSUMPTION: THE GREEN REVOLUTION

**Bao**: At the consumption end, a lot of work in energy saving and emission reduction needs to be done in various industries such as building materials, metallurgy, chemistry and transportation, touching upon many problems that need to be solved. I think the first point is that we should improve the recycling of various materials. In China, only ∼20% of aluminum is recycled, while this ratio is 80% in the US, and almost 100% in Japan. It will be a great challenge for us to systematically increase our capacity for recycling aluminum, steel and other materials. Currently, low-carbon transformations are happening in many industries. Prof. Xie may give more information from the chemical industry and other process industries.

**Xie**: China has a strong manufacturing industry. It is a foundation of national development, but it also generates a considerable amount of carbon emissions. How can process industries save energy and reduce carbon emissions? As Prof. Bao said, the first thing we can do is recycle. Especially in the iron and steel industry, developed countries have shown that mini-mills using steel scraps as raw materials can drastically reduce carbon emissions.

Another important approach is to fundamentally change industrial processes, and this involves many fields and fundamental issues. Recently, many researchers have been talking about establishing a hydrogen-based industrial system. Hydrogen has a lot of potential in industries such as petrochemical, metallurgy and building materials. In the petrochemical industry, hydrogen is mainly used to produce oils, reflecting the nature of energy. It can also be used to produce various chemicals, reflecting the nature of material. In the metallurgy and steel industry, the current major steelmaking process is to reduce iron ore with coke, which generates both steel and carbon dioxide. But now we are trying to reduce iron ore with hydrogen, which will not generate carbon emissions. In the production process of cement and other building materials, the degradation of calcium carbonate generates a great deal of carbon dioxide, and people are wondering if we can use hydrogen to react with the carbon dioxide in order to avoid carbon emissions and produce useful chemicals, such as methanol or gasoline.

We should improve the recycling of various materials.

—Xinhe Bao

However, all these hydrogen-based processes touch upon the same question: where does the hydrogen come from? Traditionally, we produce hydrogen via coal gasification and hydrocarbon reforming, but that process also generates carbon dioxide. Thus, one of the keys is to find green hydrogen sources. This task requires scientific and technological innovations to promote the development of photo/electrocatalytic splitting of water to hydrogen by using renewable energy, so as to achieve large-scale production of low-cost and clean hydrogen. Further, it will open the door to a new hydrogen-based industrial system, and create new industrial routes and systems. At present, hydrogen production technologies using renewable energy are making positive progress and laying a good foundation for breakthroughs in hydrogen-based industrial processes.

**He** **:** Waste heat utilization is another method to save energy. We can collect the waste heat generated from steelmaking or printing and dyeing and reuse it in other industrial processes. This process involves three steps: heat collection, heat storage and heat reutilization. Waste heat is not easy to collect in many cases, and the collected energy is usually not enough for direct reuse, so we need to store it first and then use it after accumulation. Furthermore, waste heat is usually contaminated, so antifouling and decontamination technologies are needed in the storage and utilization process.

Many researchers are talking about establishing a hydrogen-based industrial system.

—Zaiku Xie

**Bao**: Transportation is another important end point of energy consumption. Prof. Ouyang, would you please introduce the development of new energy vehicles, including electric vehicles and fuel cell vehicles?

**Ouyang:** Similar to the photovoltaic industry, the electric vehicle industry started to take off at the beginning of this century and has already achieved great success. The performance of household electric cars, including range, price, safety and service life, is now comparable to that of fuel cars. Sales of electric cars are fast increasing. In our estimation, electric car ownership in China will reach 30 million in 2025, 0.1 billion in 2030 and 0.3 billion in 2040, replacing most fuel cars. Accordingly, carbon emissions of household cars in China will peak around 2025, and emissions of trucks will peak around 2030.

The core technology of electric vehicles is the power battery. This is an active and fast developing innovative field. China's battery industry is at the forefront of the global industry, producing 70% of vehicle batteries worldwide. Recently, prices of raw materials have risen because of large demand. But we believe it to be temporary, and it will return to normal within two or three years. Moreover, batteries are recyclable. We estimate that by 2025, battery recycling will grow into a large-scale industry.

The development of electric vehicles is also driving the electrification of the whole transportation industry—electric boats and planes are also catching up. Moreover, it is also promoting energy storage technology and aiding the development of the entire new energy power system. I would like to introduce the concept of ‘Vehicle to Grid’ (V2G). In 2040, we will have 0.3 billion electric cars in China. With 70 kWh of electric power stored in each car, there will be 21 billion kWh of electric energy in these cars in total. For 90% of the time, the cars are just sitting there. According to our calculations, at least half of the electric energy can be redirected to maintain the stability of the power grid. In other words, electric cars will act as powerful distributed energy-storage devices. In our estimation, the demand of energy storage in China will be ∼1.8 billion kWh in 2030, which is a small number compared to that of the energy stored in the cars. Therefore, V2G has great application potential.

The fuel cell vehicle is another type of new energy vehicle. Now, the cost of fuel cell vehicles is dropping rapidly, and the industrial chain has been built in China. During the 2022 Beijing Winter Olympics, our group led the hydrogen energy demonstration project. We fulfilled the transportation demand of the games with ∼1000 hydrogen fuel cell vehicles, and all the hydrogen was produced by renewable energy. After the Olympics, more fuel cell vehicle demonstration projects began in several cities in China. In our estimation, the ownership of fuel cell vehicles will rise to 50 000–100 000 in China by 2025.

Furthermore, the development of hydrogen fuel cells will drive the development of the entire hydrogen industry by its accumulation of experience with regard to the industrial chain, which consists of hydrogen production, storage and usage. In order to achieve carbon neutrality, China needs to produce >80 million tons of hydrogen per year, and 70%–80% of the hydrogen should be produced by green energy. The technologies used in fuel cells will also promote the technologies of hydrogen production by water electrolysis, because these two processes are reverse processes of each other and share the same industrial chain. About hydrogen usage, as Prof. Xie said, hydrogen will play an important role in metallurgy, chemical industry and many other fields. Also, we can burn hydrogen to generate electricity—a cleaner substitute for coal-fired plants—in order to stabilize the future renewable power system.

So, we can see that new energy vehicles, including electric vehicles and fuel cell vehicles, will not only reduce the carbon emissions of the transportation system, but will also drive the green transformation of the entire power and industrial system.

New energy vehicles, including electric vehicles and fuel cell vehicles, will not only reduce the carbon emissions of the transportation system, but will also drive the green transformation of the entire power and industrial system.

—Minggao Ouyang

## ENERGY STORAGE: CENTRALIZED AND DISTRIBUTED

**Bao**: Energy storage technology is critical for the renewable-energy-based new energy system. What are the current trends in this field?

**Cheng**: Both photovoltaic and wind energy are intermittent and unstable, so the application of renewable power cannot be realized without energy storage technologies. There are different kinds of energy storage technologies that can transfer intermittent energy into continuous energy. Specifically, we need both large-scale centralized energy storage and small-scale distributed energy storage.

Large-scale energy storage is mostly used at the power-generation end, coupled with large-scale power plants. Currently, the most powerful large-scale storage method is pumped hydro storage. But the contribution of electrochemical energy storage is quickly increasing. To better apply electrochemical methods for large-scale storage, an important direction is to make better use of abundant elements, but not the rare elements such as lithium. Therefore, sodium ion batteries and zinc ion batteries may have great potential to contribute more to large-scale energy storage.

On the other hand, distributed energy storage should be coupled with distributed power generation, as Professor Shi said. The electrochemical method is the major force of distributed energy storage—energy storage in electric vehicles, as mentioned by Prof. Ouyang, is an example of it. Some other methods, such as phase change energy storage and other lithium-free energy storage technologies can also be used for distributed energy storage.

Large-scale energy storage is mostly used at the power-generation end, coupled with large-scale power plants.

—Huiming Cheng

## CARBON SEQUESTRATION: THE INSURANCE OF CARBON NEUTRALITY?

**Bao**: Even with all our efforts to reduce emissions, they will not be reduced to zero as long as we still use some fossil fuels. Therefore, to achieve carbon neutrality, we need carbon capture, utilization and storage (CCUS) technologies to capture and store the extra carbon. But there seems to be much debate about the readiness, cost and capacity of these technologies. Would Prof. Guo give us some thoughts on this issue?

**Guo**: According to the current research, with all the emission reduction measures implemented, there will still be 2–3 billion tons of carbon dioxide emissions left to be stored by measures beyond emission reductions in 2060. It is widely believed that ecological carbon sequestration and CCUS technologies should be utilized to solve the problem.

We can try to explore and accelerate all the natural processes that can sequester carbon.

—Zhengtang Guo

The carbon sink over terrestrial ecosystems in our country has attracted the most attention recently. Through ecological engineering and ecological optimization, it is estimated that the terrestrial ecosystems in China can absorb ∼1 billion tons of carbon dioxide per year. But since the carbon sink capacity of terrestrial ecosystems will gradually come to saturation, our understanding of its role for the 2060s remains to be clarified. Therefore, I think we should treat enhancement of land carbon sinks through ecological engineering as a low-cost measure to go through the window period of carbon neutrality. It will help buy us some more time to further reduce emissions. Furthermore, ecological engineering should be implemented with a carefully designed time plan—if it starts too early, the land carbon sink may saturate well before 2060, which could mean it fails to support the carbon neutrality goal in 2060.

The ocean is an important part of the ecological carbon sink. It is the biggest carbon pool on Earth. The loss or gain of minor fractions of the ocean carbon pool can result in huge variations of atmospheric carbon dioxide concentration. However, the ocean system is extremely complex, and we have not understood what the ecological consequence of human intervention would be, so there are currently few efforts targeting the enhancement of the ocean carbon sink. But considering its importance, there is an urgent need to find possible methods of ocean engineering. For example, maybe we can regulate marine microorganisms to absorb more carbon. And practically, we can further study the offshore marine systems that have already been deeply affected by human activities, in order to protect and restore the carbon sink capacity of these ecosystems.

Besides the ecosystem carbon sink, several CCUS methods have been proposed. In China, the most practical method is carbon dioxide flooding technology, which injects carbon dioxide into the oil and gas wells, pressing oil and gas out and sequestering carbon dioxide underground at the same time. Recently, some foreign researchers proposed other potential strategies that make use of the carbon capture capacity of marsh peat, silicate weathering, estuarine carbonate precipitation, basins in arid regions and the groundwater in karst areas. Some researchers believe that these methods may have great carbon capture capacity. For example, there is a large amount of ultrabasic rock on the Earth; if we can use carbon dioxide to carbonize this rock, the carbon dioxide will be solidified into calcium carbonate and get stored there almost permanently. In summary, we can try to explore and accelerate all the natural processes that can sequester carbon. But these studies are just beginning, and in China, especially, are at the nascent stage.

Another important issue worth mentioning is the stability of sequestered carbon. This is indeed relevant to a basic scientific question on how climate change interacts with the carbon cycle. The Earth's carbon cycle is in a dynamic equilibrium, similar to buffer systems in chemical experiments. If we artificially sequester a lot of carbon, the terrestrial and marine ecosystems may store less or even release more carbon, which would offset our efforts. Some researchers estimated that the reduction of atmospheric CO_2_ concentration would be equivalent to ∼20% of the anthropogenic removal of carbon dioxide. The remaining 80% will eventually be released by the ecosystems. With this in mind, we should carefully study this issue and look for more stable CCUS methods, such as the various geological carbon sinks.

**Bao**: Thanks for the introduction. It seems that we need to do much more work on the issues of carbon sequestration. Some of the problems are related to chemistry and may need the efforts of chemists and researchers from other disciplines.

In conclusion, I would like to thank you for all your insights. The target of carbon neutrality will drive a system-wide green revolution of China. We talked about power generation, which needs the development of renewable energy and more efficient use of fossil fuels. We talked about the energy-consumption industries, touching on materials recycling, waste heat utilization, new energy vehicles and the prospect of building a hydrogen-based industrial system. For energy storage technology, we emphasized the use of distributed energy storage such as electric vehicles, and also called for the continued development of centralized electrochemical energy storage methods such as sodium-ion batteries. We also talked about carbon-sequestration methods that need to be further explored. All in all, we should mobilize our entire society to make a concerted effort to save energy and reduce carbon emissions. Our collective goal is to achieve carbon neutrality and protect the ecological safety of our planet.

## Notes

Weijie Zhao is an NSR news editor based in Beijing, China.

